# Fatigue in ferry shipping employees: the role of work-family conflict and supervisor support

**DOI:** 10.1186/s12889-019-7954-z

**Published:** 2019-12-17

**Authors:** Solveig Boeggild Dohrmann, Kimmo Herttua, Anja Leppin

**Affiliations:** 1 Department of Public Health, Centre of Maritime Health and Society, University of Southern Denmark, Niels Bohrs Vej 9, 6700 Esbjerg, Denmark; 2 Department of Public Health, Unit of Health Promotion Research, University of Southern Denmark, Niels Bohrs Vej 9, 6700 Esbjerg, Denmark

## Abstract

**Background:**

Fatigue is a concern in ferry shipping as it has a negative impact on crew members health and plays a major role in marine incidents and accidents. Research within land-based occupational settings has found that work-family conflict is an important risk factor for fatigue and that support from leaders constitutes a possible resource with the potential to buffer a negative impact from work-family conflict. Though, the working conditions of ferry shipping are likely to interfere with employee’s family life those two factors have received little attention in research on seafarers’ health. Therefore, the aim of this study was to investigate the direct associations between work-family conflict as well as leaders’ support with fatigue in employees of the Danish ferry shipping industry. Further, the study aimed at testing whether support could buffer potential detrimental associations between work-family conflict and fatigue.

**Methods:**

The study design was cross-sectional, and 193 respondents answered to a self-administered questionnaire. Fatigue was measured with the Swedish Occupational Fatigue Inventory. Perceived work-family conflict and perceived supervisor support were assessed with two subscales from the Copenhagen Psychosocial Questionnaire. The association of potential risk factors with fatigue was determined using hierarchical multiple linear regression analyses.

**Results:**

After controlling for confounding, work-family conflict was found to be positively associated with four of the five subdimensions of fatigue; lack of energy, physical discomfort, lack of motivation and sleepiness, while more support from supervisors was related to less lack of energy, physical exhaustion and lack of motivation. Further, supervisor support was found to moderate the effect from work-family conflict on the physical subdimensions of fatigue.

**Conclusion:**

Although restricted by its cross-sectional design and a limited sample, this study provides support for the independent relevance of work family conflict and support from nearest superior for employee fatigue in ferry shipping. Further, there was evidence for a moderating role of such support on the negative impact of work-family conflict on the physical aspects of fatigue. Shipping companies may consider commencing initiatives which reduce conflicts between family life and work obligations, and that leader support may be a relevant component in such initiatives.

## Background

Seafaring is considered a challenging occupation, involving long and irregular working hours, not rarely in inclement weather, working non-standard and shifting work times including nights, as well as – for many -extended periods away from home. Further, like many other industries, seafaring has become a highly competitive 24 h-business characterized by an increasing workload to be dealt with under constant time pressure [1, 2]. Conditions such as these are likely to contribute to subjective stress experience, including fatigue, disrupted sleep, and poor sleep quality on board [3, 4].

In onshore occupational settings, estimates for reported fatigue among employees vary between 12 and 38% [5–7], whereas proportions from 38 to 76% have been found in seafaring populations [8–10]. More specifically, as many as 89% of crew members reported to have lost concentration while at work due to fatigue, up till 33% have been involved in a fatigue-related incidence or accident, and 23% have fallen asleep at work more than once over a month [11, 12]. Fatigue has been found to be negatively associated with individual health and well-being as well as work ability across industries [13–15]. Furthermore, in seafaring, just as in other branches of the transport industry, public safety is an important additional concern [16]. In fact, seafarers’ fatigue has recurrently been identified as either the primary cause or a key contributing factor to marine incidents and accidents [17], endangering the safety of the crew, the ship and the environment [18]. In ferry shipping, fatigue is particularly problematic, as it may also put passengers at risk [12, 19].

While fatigue has thus been identified as a potential problem to tackle by the maritime industry, more knowledge is needed to launch systematic preventive efforts [3, 4]. One such challenge is linked to the often oversimplified conceptualization and measurement of the construct “fatigue”. In an occupational context, fatigue is usually described as a sense of extensive tired- or sleepiness and is mostly perceived of as a more acute condition, which however can accumulate over time towards a more chronic physical and mental impairment [16]. Occupational fatigue is caused by physical and/or mental exertion at work, such as tight and/or unexpected deadlines, new tasks or overtime and is reversible by means of sufficient rest [13, 16]. Occupational fatigue thus results from specific work tasks which differ in terms of what kind of physical and/or mental demands they impose on the employee in different work environments and which types of symptoms they cause [16]. Multi-dimensional assessment of fatigue symptoms may help identify such specific patterns and thereby provide deeper insights into which aspects of fatigue specifically need to be targeted by preventive and treatment interventions [16, 20]. So far, however, occupational fatigue has mostly been assessed by single item measures [3–5, 20].

An exception is the approach suggested by Ahsberg et al. [21] and Ahsberg [22] who have proposed five dimensions of work-related fatigue, i.e. lack of energy, physical exertion, physical discomfort, lack of motivation and sleepiness, and who have identified different fatigue profiles for different land-based occupational groups, such as bus drivers, locomotive engineers, firemen, workers in factories and teachers with varying types of work tasks. However, so far only one study has investigated such subdimensions of fatigue in seafaring occupations. Thus, Leung et al. [23] used the Chinese version of the Swedish Occupational Fatigue Inventory to examine the fatigue experienced by high speed maritime craft officers who worked either a day or a night shift. Results showed that both day- and night-shift officers experienced fatigue as they worked through a shift. Higher levels - and a more rapid rate in increase of fatigue during a shift, however, were found among officers working night shifts. Furthermore though, physical fatigue, i.e. physical exertion and physical discomfort, was found to be especially high in the group of night-shift officers while this was not the case for mental fatigue, i.e. lack of energy, lack of motivation and sleepiness [23]. These differential findings demonstrate a need for further investigations of such subdimensions of fatigue in seafaring occupations.

A second challenge relates to the fact that research on determinants of fatigue has been limited in the maritime setting, focusing mostly on the physical environment and the role of shift /night work [3, 4, 24, 25]. This contrasts with general occupational health research, where there has been a growing focus on the psychosocial work environment and its impact on workers’ mental health, including stress and fatigue [26–30], while only a few studies so far have provided evidence for the role of psychosocial work demands on fatigue in the seafaring domain [3, 4].

Psychosocial factors can be divided into originating from work demands (e.g. workload and work pace), from interpersonal relations (e.g. cooperation between colleagues), from leadership (e.g. leader support), from values at the workplace (e.g. justice) [31] as well as from the work-individual interface (e.g. work-family conflict. From a predominant focus on job demands and job control in the early years [32], research has developed towards investigating a wide range of factors from all five domains [26, 27, 31] as well as the relation *between* factors [33–36]. Thus, for instance, in more recent years the work-individual interface and leadership have attracted much research interest, and some studies have indicated that conflicts between work and family demands which jeopardize (mental) health could be increasing but that support from leaders may be a relevant mitigating factor [26, 33–36].

According to role theory most human activities are to be considered an acting out of socially defined roles, such as ‘employee’ and ‘parent’ [37]. Many employees face the challenge of combining work and family roles, which can result in a role conflict – a situation in which an individual is faced with incompatible demands from work and family domains of life, making it difficult to enact both roles satisfactorily [37–39]. For many land-based industries it has been shown that work demands intrude into employees’ family life [37–39], and that ensuing conflicts may lead to negative mental health outcomes [39], sleep disturbances [40] and fatigue [41, 42] among employees. Ferry shipping, in particular, is characterised by tight time tables and an ever-urgent need for safe and timely delivery of passengers during day-, evening- and night-times on all days of the week [1]. Such working conditions are very likely to interfere with employees’ family life [37–39] - hereby putting the employees at increased risk for experience of fatigue. This remains speculative, however, since surprisingly, no research has yet been conducted in the maritime occupational field.

In land-based occupations it has been shown that availability of resources is able to counteract a potential negative impact of work stress, either by having direct protective effects on health outcomes or by buffering detrimental effects of stress [43–45]. Thus, research including meta-analyses, has shown that such support is a relevant resource in successfully combining demands of work and family life [27–30, 33–36, 46]. Specific helpful interventions in challenging situations as well as a general sense of being supported by one’s supervisor have been found not only to influence employee’s level of fatigue [7, 42, 47] and sleep quality [48], but also, as a consequence, job satisfaction, job well-being and going on disability pension [47].

In a maritime context and in ferry shipping in particular, supervisor support might prevent the development of work-family conflict [1, 2, 25] or buffer potential negative health consequences for instance, when it comes to negotiating the adjustment of tight work schedules to family emergencies or suddenly occurring needs to take care of family obligations [37, 39]. Further, like in other occupations, it might boost a general sense of being appreciated [28–30] and thereby directly counteract mental exhaustion [7, 42, 47]. However, the role of supervisor support has yet to be investigated in a maritime context.

In view of the outlined gaps in evidence about the role of work-family conflict and supervisor support for fatigue in seafaring/ferry shipping, the present study aimed to investigate

- whether there are direct main associations between perceived levels of work-family conflict, support from nearest superior and different dimensions of fatigue;
- whether there is an interaction between the stress factor ‘work-family conflict’ and the social resource ‘supervisor support’ on fatigue dimensions among employees in Danish ferry shipping after controlling for the influence from general job demands as well as sleep quality.

## Methods

The present study used a cross-sectional survey design, and data collection was based on a standardized questionnaire battery. For reporting, the checklist ‘Strengthening the Reporting of Observational Studies in Epidemiology’ was used for guidance [49].

### Participants and procedure

Participants were recruited mainly from one Danish ferry ship company operating a total of five domestic and two international services, all of which were included in the study (Company 1). In addition, we collected data from one further Danish ferry shipping company (Company 2). This company operated three international services, one of which was included in the study. Data were collected from April to the end of September 2015. In some ferry companies crew members can be expected to have a shipboard stay of several days, whereas those working in the terminals have an eight-hour work day in the same place. However, in both companies all ferry ships except one were laid up overnight. This meant that most crew members actually slept at home (though still on call during their service periods) - alternatively in onshore watch-rooms if time was too short to travel home. Thus, contextual conditions, such as schedules (including working early mornings, evenings and late nights), time away from home and sleeping conditions, were largely similar for crew members and terminal workers, which is why both groups were considered eligible for participation in the study.

All eligible employees were invited via written information. Further, we made the questionnaire available electronically as well as in a printed form. Information about the study and a link to the online questionnaire, including a description of how to get access and fill it out, was 1) made available on the companies’ intranets, and 2) sent out via email. Additionally, a paper version of the questionnaire was distributed by terminal managers and senior captains to eligible employees who preferred a printed format. Two reminders were sent. The first via email, 3 weeks after the questionnaire had been sent. The second was distributed via the terminal managers and senior captains another 3 weeks after.

The questionnaire was distributed to a total of 513 employees; Company 1 = 281 and Company 2 = 232 (179 terminal workers and 334 crew members). One hundred ninety three returned a completed questionnaire yielding a response rate of 56 and 16%, respectively. The sample characteristics are shown in Table 1.

**Table 1:** Characteristics of the study population and the working environment

	*n*	%	Mean	SD
Age	172		47. 6	12.4
Gender (female)	19	11		
School education				
≥ 7th grade - technical school	90	57		
Gymnasium education	68	43		
Professional education				
Vocational training	77	45		
University college and university	94	55		
Professional group				
Officers	102	53		
Non-officers	91	47		
Living with a partner (yes)	135	78		
Children under six years old (yes)	25	15		
Ferry ship company				
Company 1	156	81		
Company 2	37	19		
Workplace				
Terminal	34	18		
Ferry ship	158	82		
Number of workdays per week	179		3.85	0.90
Typical time of work				
Day and evening	96	52		
Day, evening and night	89	48		
Sleeping at the workplace/on board (yes)	72	45		
Physical activity				
0–4 h per week. Low intensity	53	31		
≥ 2 h per week. High intensity	119	69		
Smoking (yes)	43	25		
Sleeping troubles (0 (low)-25–50–75-100 (high))	176		31.39	19.55
Job demands (0 (low)-25–50–75-100 (high))	184		48.86	14.18
Work-family conflict (0 (low)-25–50–75-100 (high))	192		32.29	21.55
Support from supervisor (0 (low)-25–50–75-100 (high))	186		55.82	20.09
SOFI Lack of energy (0 (very low)–6 (very high))	166		2.02	1.55
SOFI Physical exertion (0 (very low)–6 (very high))	166		1.21	1.31
SOFI Physical discomfort (0 (very low)–6 (very high))	166		1.48	1.43
SOFI Lack of motivation (0 (very low) – 6 (very high))	166		1.56	1.45
SOFI Sleepiness (0 (very low)– 6 (very high))	166		1.79	1.40

The study was approved by the Danish Data Protection Agency (Datatilsynet) [50]. According to Danish law at the time the study was conducted, questionnaire surveys like the present one did not need approval from The Regional Ethical Committees of Southern Denmark (De Videnskabsetiske Komiteer for Region Syddanmark) (§ 14) [51]. Further, and in accordance with existing law, consent to participate was given by ‘explicit enactment’, i.e. by submitting the completed questionnaire (§ 3) [52].

### Measurement

#### Outcomes

The second, revised version of the Swedish Occupational Fatigue Inventory (SOFI) [22] was used to measure fatigue. We translated the English version into Danish using translation/back-translation technique [53]. SOFI includes 20 items which are divided into five subscales with four items each: lack of energy (LE), physical exertion (PE), physical discomfort (PD), lack of motivation (LM) and sleepiness (S). All items are consisting of one attribute that describes fatigue-related feelings or symptoms, such as ‘worn out’ (LE), ‘palpitations’ (PE), tense muscles’ (PD), ‘lack of concern’ (LM) and ‘falling asleep’ (S). Respondents are to rate items on a response scale from 0 = ‘not at all’ to 6 = ‘to a very high degree’ with regard to how they felt when they were most tired when at work during the last 4 weeks. For each subscale, a sum score was calculated, indicating the symptom-specific perceived level of fatigue [22]. Internal consistencies for the subscales were α = .87 (LE), α = .86 (PE), α = .86 (PD), α = .93 (LM) and α = .93 (S), respectively.

#### Exposures

We assessed the two domains of exposure by two subscales from the second version of the Copenhagen Psychosocial Questionnaire (COPSOQ). COPSOQ is a standardized and validated questionnaire that covers a wide range of psychosocial work environment factors [31].

The ‘work-family-conflict’ - and ‘supervisor support’ scales consist of four and three items, respectively. The first scale measures thoughts and feelings related to work-family balance, including items such as ‘Do your friends and family tell you that you work too much?’. The latter measures perceptions of supportive behaviors from nearest superior, reflected in items such as ‘How often do you get help and support from your nearest supervisor?’. Four-point response scales tapping degree of certainty and frequency (1 = ‘no, not at all’ to 4 = ‘yes, certainly’ and 1 = ‘no, never’ to 4 = ‘yes, often’) accompany the work-family conflict items. The supervisor support items are to be rated on five-point scales measuring perceived intensity (1 = ‘to a very small extent’ to 5 = ‘to a very large extent’). In accordance with guidelines, we rescored all items (0–100, i.e. 1 = 0, 2 = 25, 3 = 50, 4 = 75 and 5 = 100) and calculated a sum-score [31]. Cronbach alpha coeffients were α = .74 for ‘work-family conflict’ and α = .69 for ‘support from nearest superior’. To be able to test the interaction between work-family conflict and supervisor support the two variables were first centered at the mean and then multiplied.

#### Covariates

Socio-demographic factors, such as country of birth, age, gender, school (primary−/secondary level), vocational education (vocational training/university college or university) and living with a partner, were considered as standard covariates, and single-item measures were used in their assessment.

The selection of further potential confounders was based on theoretical assumptions about possible confounding factors for the work-family conflict-, supervisor support- and fatigue-relationship as well as empirical evidence for associations documented in four reviews on seafarers’ stress and fatigue [1–4]. On this basis, we chose to test - on the bivariate level - work-related factors, i.e. professional group (officers/non-officers), ferry ship company (company 1/company 2), workplace (ferry/terminal), number of workdays per week, sleeping at the workplace (yes/no), and night work (no night work/night work) indicating shift work. Also, the lifestyle-related factors smoking status (no/yes) [54] and physical activity (low intensity/high intensity) were tested since they have been found to influence occupational fatigue [54, 55].

Further, since fatigue is bound to be influenced by sleep quality [1–4], and sleep quality is likely to be negatively affected for employees working night shifts or sleeping at the workplace during their shifts, we decided to also test this factor. Sleep quality was assessed by a four-item scale from the COPSOQ [31]. Items were rescored and a sum score was computed [31]. Cronbachs’ alpha coefficient was α = .85.

Finally, we also tested an effect of job demands. Occurrence of conflict between work and family life are likely to increase with higher job demands [33, 35]. At the same time, the level of job demands has been related to fatigue in a seafaring context [1–4]. To identify a potential specific impact of work-family conflict, partialling out the effect of job demands therefore seemed important. For rmeasurement we again used COPSOQ [31], i.e. the two subscales ‘quantitative demands’ (four items) and ‘work pace’ (three items) [31]. Items were rescored, and a sum-score of both subscales was calculated [31]. Cronbachs’ alpha coefficient was α = .77.

### Data analysis

When summing up scores for the COPSOQ and SOFI subscales, missing values were replaced by individual subscale means in accordance with guidelines (COPSOQ: the work-family conflict subscale was complete and two missing values were replaced within the supervisor support subscale. SOFI: LE, PD, and S were complete, and one and two missing values were replaced within PE and LM, respectively) [22, 31]. We used Pearson correlations to first determine bivariate associations between scores of each outcome variable and potentially associated variables.

Multivariable analyses were conducted using hierarchical multiple regressions. To control for potential confounding but at the same time maximize power, only age, gender and those personal and job/work-place characteristics which on the bivariate level had been significantly associated with one of the outcome variables (*p* < .05), were entered on step one of each of the five models. An exception to this was ‘professional education’. As expected, this variable was highly correlated with ‘professional group’, i.e. being an officer versus non-officer. As the latter characteristic seemed more important to the outcomes under study [1–4], ‘professional education’ was deselected to avoid multicollinearity. Sleeping problems were entered in step two and work-family conflict as well as supervisor support in step three. The work family conflict-supervisor support interaction was added in the fourth and last step. The simple slope technique was used to further explore any interactions [56]. Age, sleeping troubles and all psychosocial work factors were used as continuous variables, whereas all other characteristics were entered as binary variables.

Prior to the analysis, tests were run to check for potential violations of assumptions in terms of linearity, multivariate normality, homoscedasticity, multicollinearity and outliers. In the process of outlier identification, between zero and seven multivariate outliers for the five different models were detected based on Cook’s distance criterion. The identified cases were eliminated from the respective models. The remaining sample sizes ranged from *n* = 193 (S) to *n* = 186 (PE). All analyses were conducted with SPSS Statistics 24.

## Results

The large majority of the study population (98%) were of Danish origin. As can be seen in Table 1, 89% of participants were male, and mean age was 47.6 (range from 19 to 70). About 50% were officers, 82% were working on board of ferries, and for 50% of employee’s typical work time involved night shifts (Table 1).

Prior to analyses we used the subsample from company 1, which made up most of the study sample, to test whether participants in the study differed from all crew members and terminal workers in the respective company regarding age, gender, workplace (on board of a ferry/terminal) and professional group (officers/non-officers). No significant differences were found (findings available from the corresponding author).

### Bivariate associations

The correlation coefficients in Table 2 show that females and non-officers reported more physical discomfort. Further, non-officers also reported higher levels of sleepiness. In terms of work-related factors, those employed in the terminals stated higher levels of physical discomfort and sleepiness. Those with habitual night work indicated higher levels in all aspects of fatigue. Additionally, higher levels of sleeping problems, job demands, and work-family-conflicts were associated with higher levels of fatigue, while more support from nearest superior was associated with lesser fatigue.

**Table 2:** Bivariate correlations between personal characteristics, worksite characteristics, work stressors and different dimensions of fatigue

	SOFI lack of energy	SOFI physical exertion	SOFI physical discomfort	SOFI lack of motivation	SOFI sleepiness
Age	−0.02	−0.05	− 0.08	− 0.13	− 0.13
Gender^a^	0.10	0.02	0.21**	0.06	0.14
School education^b^	0.05	−0.04	0.01	0.03	0.05
Professional education^c^	−0.11	−0.06	− 0.20*	−0.12	− 0.16*
Professional group^d^	0.14	0.06	0.21**	0.13	0.18*
Living with a partner^e^	0.04	0.10	0.11	0.01	0.07
Children under 6 years^f^	−0.12	−0.06	− 0.10	−0.07	− 0.07
Ferry ship company^g^	0.09	0.02	0.13	0.04	0.10
Workplace^h^	−0.13	− 0.09	− 0.16*	−0.14	− 0.20*
Number of workdays per week	−0.08	− 0.04	0.07	− 0.13	−0.06
Typical work time^i^	0.20*	0.16*	0.26**	0.17*	0.19*
Sleeping at the workplace/on board^j^	−0.12	−0.08	−0.13	− 0.13	−0.15
Physical activity ^k^	−0.10	0.01	−0.04	−0.04	− 0.07
Smoking^l^	0.05	0.02	0.07	0.02	−0.01
Sleeping trouble	0.45′**	0.35***	0.39***	0.36***	0.44***
Job demands	0.40***	0.30***	0.27***	0,34***	0.36***
Work-family conflict	0.48***	0.33***	0.36***	0.43***	0.42***
Support from supervisor	−0.28***	− 0.19*	− 0.23**	−0.31***	− 0.26**
SOFI Lack of energy	1	0.66***	0.70***	0.80***	0.82***
SOFI Physical exertion	0.66***	1	0.67***	0.74***	0.72***
SOFI Physical discomfort	0.70***	0.67***	1	0.60***	0.67***
SOFI Lack of motivation	0.80***	0.74***	0.60***	1	0.84***
SOFI Sleepiness	0.82***	0.72***	0.67***	0.84***	1

*Significant value: *P* <0.05. ** Significant value: *P* <0.01. ***Significant value: *P* <0.001.

^a^female = 2

^b^primary school = 1, secondary school = 2

^c^vocational training = 1, university college/university = 2

^d^officers = 1, non-officers = 2

^e^yes = 2

^f^yes = 2

^g^company 1 = 1, company 2 = 2

^h^terminal = 1, ferry ship = 2

^i^day and evening = 1, day, evening and night = 2

^j^yes = 2

^k^0-4 h per week/low intensity = 1, ≥2 hoursh per week/high intensity = 2

^l^yes = 2

### Multivariable associations

Results from the analyses are presented in Tables 3, 4, 5, 6 and 7. As can be seen, adding the main predictors revealed a significant contribution from work-family conflict to four of the five aspects of fatigue after controlling for potential confounders, while supervisor support was significantly associated with three of the fatigue sub-dimensions (Tables 3, 4, 5, 6 and 7, model 3). Model 4 shows that higher levels of perceived work-family conflict went along with higher levels of lack of energy (β = .35) (Table 3), physical discomfort (β = .21) (Table 5), lack of motivation (β = .29) ( Table 6) and sleepiness (β = .25) (Table 7). Further, model 4 shows that higher levels of supervisor support were associated with lesser lack of energy (β = −.13) (Table 3), physical exertion (β = −.18) (Table 4) and lack of motivation (β = −.27) (Table 6). Furthermore, the work-family conflict-supervisor support interaction term was found to be significant for the two physical sub-dimensions of fatigue, i.e. physical exertion (β = −.25) and physical discomfort (β = −.19) (Table 4, Table 5, model 4). As support from supervisor may attenuate experience of work-family conflict, these two factors may not be independent, which might make an interaction term problematic. However, the bivariate association was only moderate (r = −.27), which allowed for inclusion of an additional interaction term.

**Table 3:** Multivariable associations between work-family conflict, supervisor support and lack of energy

SOFI Lack of energy (*n* = 156)												
	Model 1			Model 2			Model 3			Model 4		
	β	R^2^	R^2^ change	β	R^2^	R^2^ change	β	R^2^	R^2^ change	β	R^2^	R^2^ change
Age	0.10			0.13			0.20**			0.20**		
Gender^a^	0.10			0.08			0.11			0.11		
Workplace^b^	−0.05			−0.05			0.02			0.02		
Professional group^c^	0.11			0.08			0.16*			0.15*		
Typical time of work^d^	0.14			0.04			−0.03			−0.03		
Job demands	0.40***			0.32***			0.17*			0.17*		
		0.23***										
Sleeping troubles				0.37***			0.27***			0.27***		
					0.35***	0.11***						
Work-family conflict							0.36***			0.35***		
Support from supervisor							−0.13*			−0.13*		
								0.46***	0.11***			
Conflict*super-visor support										0.04		
											0.46***	0.00

β – Standardized regression coefficient *Significant value: <0.05 ** Significant value: <0.01 ***Significant value: <0.001

^a^Female = 2. ^b^Terminal = 1, Ferry ship = 2. ^c^Officers = 1, Non-officers = 2. ^d^Day and Evening = 1, Day, Evening and Night = 2

**Table 4:** Multivariable associations between work-family conflict, supervisor support and physical exertion

SOFI Physical exertion (*n* = 152)												
	Model 1			Model 2			Model 3			Model 4		
	β	R^2^	R^2^ change	β	R^2^	R^2^ change	β	R^2^	R^2^ change	β	R^2^	R^2^ change
Age	− 0.02			−0.02			0.02			0.03		
Gender^a^	− 0.06			−0.06			−0.05*			−0.09		
Workplace^b^	−0.14			−0.15			−0.09			−0.10		
Professional group^c^	−0.03			−0.06			−0.02*			−0.07		
Typical time of work^d^	0.19*			0.11			0.06			0.06		
Job demands	0.27**			0.20*			0.11			0.11		
		0.13**										
Sleeping troubles				0.31***			0.23**			0.23**		
					0.21***	0.08***						
Work-family conflict							0.16			0.12		
Support from supervisor							−0.18*			−0.18*		
								0.26***	0.05**			
Conflict*super-visor support										−0.25**		
											0.32***	0.06**

β – Standardized regression coefficient *Significant value: <0.05 ** Significant value: <0.01 ***Significant value: <0.001

^a^Female = 2. ^b^Terminal = 1, Ferry ship = 2. ^c^Officers = 1, Non-officers = 2. ^d^Day and Evening = 1, Day, Evening and Night = 2

**Table 5:** Multivariable associations between work-family conflict, supervisor support and physical discomfort

SOFI Physical discomfort (*n* = 157)												
	Model 1			Model 2			Model 3			Model 4		
	β	R^2^	R^2^ change	β	R^2^	R^2^ change	β	R^2^	R^2^ change	β	R^2^	R^2^ change
Age	0.03			0.05			0.09			0.10		
Gender^a^	0.18*			0.16*			0.18*			0.15*		
Workplace^b^	−0.03			−0.03			0.02			0.01		
Professional group^c^	0.16			0.14			0.19*			0.15		
Typical time of work^d^	0.23**			0.14			0.09			0.09		
Job demands	0.23**			0.16*			0.05			0.05		
		0.20***										
Sleeping troubles				0.30***			0.22**			0.22**		
					0.28***	0.08***						
Work-family conflict							0.24**			0.21*		
Support from supervisor							−0.12			−0.12		
								0.34***	0.06**			
Conflict*super-visor support										−0.19**		
											0.37***	0.03**

β – Standardized regression coefficient *Significant value: <0.05 ** Significant value: <0.01 ***Significant value: <0.001

^a^Female = 2. ^b^Terminal = 1, Ferry ship = 2. ^c^Officers = 1, Non-officers = 2. ^d^Day and Evening = 1, Day, Evening and Night = 2

**Table 6:** Multivariable associations between work-family conflict, supervisor support and lack of motivation

SOFI Lack of motivation (*n* = 154)												
	Model 1			Model 2			Model 3			Model 4		
	β	R^2^	R^2^ change	β	R^2^	R^2^ change	β	R^2^	R^2^ change	β	R^2^	R^2^ change
Age	−0.07			−0.04			0.01			0.01		
Gender^a^	−0.03			−0.03*			−0.02			−0.03		
Workplace^b^	−0.09			−0.09			−0.01			−0.01		
Professional group^c^	0.04			0.02			0.08			0.06		
Typical time of work^d^	0.21**			0.14			0.06			0.06		
Job demands	0.35***			0.29***			0.14			0.14		
		0.21***										
Sleeping troubles				0.28***			0.16*			0.16*		
					0.27***	0.07***						
Work-family conflict							0.31***			0.29***		
Support from supervisor							−0.27***			−0.27***		
								0.41***	0.14***			
Conflict*super-visor support										−0.10		
											0.41***	0.01

β – Standardized regression coefficient *Significant value: <0.05 ** Significant value: <0.01 ***Significant value: <0.001

^a^Female = 2. ^b^Terminal = 1, Ferry ship = 2. ^c^Officers = 1, Non-officers = 2. ^d^Day and Evening = 1, Day, Evening and Night = 2

**Table 7:** Multivariable associations between work-family conflict, supervisor support and sleepiness

SOFI Sleepiness (*n* = 159)												
	Model 1			Model 2			Model 3			Model 4		
	β	R^2^	R^2^ change	β	R^2^	R^2^ change	β	R^2^	R^2^ change	β	R^2^	R^2^ change
Age	−0.08			−0.05			−0.01			−0.01		
Gender^a^	0.01			−0.01			0.01			0.01		
Workplace^b^	−0.17			− 0.17*			−0.13			−0.13		
Professional group^c^	0.11			0.09			0.14			0.14		
Typical time of work^d^	0.12			0.03			−0.02			−0.02		
Job demands	0.34***			0.27***			0.16*			0.16*		
		0.20***										
Sleeping troubles				0.32***			0.25**			0.25**		
					0.29***	0.09***						
Work-family conflict							0.26**			0.25**		
Support from supervisor							−0.10			−0.10		
								0.35***	0.06**			
Conflict*super-visor support										−0.04		
											0.35***	0.00

β – Standardized regression coefficient *Significant value: <0.05 ** Significant value: <0.01 ***Significant value: <0.001

^a^Female = 2. ^b^Terminal = 1, Ferry ship = 2. ^c^Officers = 1, Non-officers = 2. ^d^Day and Evening = 1, Day, Evening and Night = 2

**Fig. 1:**
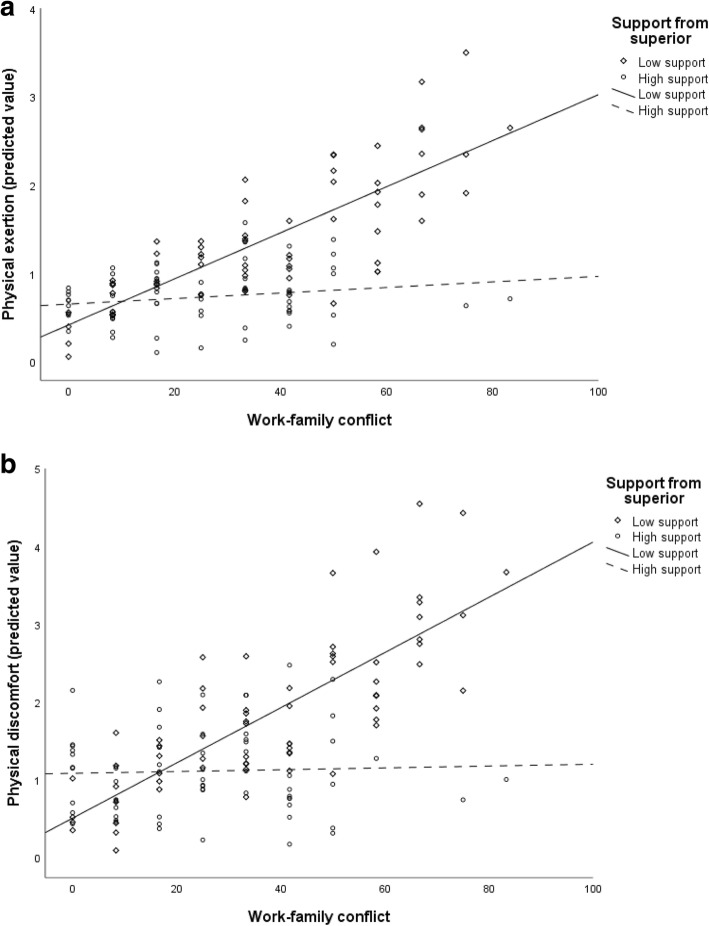
**a** Moderating effect of supervisor support on the work-family conflict - physical exertion association. **b** Moderating effect of supervisor support on the work-family conflict - physical discomfort association

Results from the simple slope regression models revealed that when supervisor support was low, perceived work-family conflict was significantly associated with both physical exertion (β = .36, *p* < .001) and physical discomfort (β = .40, p < .001). In contrast, there was no significant relation between work-family conflict and either of the two dimensions of fatigue at high levels of support (physical exertion: β = −.12, *p* = .33 and physical discomfort: β = .03, *p* = .783). This indicates that perception of high supervisor support was buffering the effects from work-family conflict on physical exertion and physical discomfort but not those for the mental sub-dimensions of fatigue. The interactions are illustrated in Fig. 1a (physical exertion) and Fig. 1b (physical discomfort).

The amount of variance explained by the final regression models ranged from 32% for physical exertion (Table 4, model 4) to 46% for lack of energy (Table 3, model 4). Sleeping troubles were found to be a key factor explaining variance from 21% (physical exertion) (Table 4, model 2) to 35% (lack of energy) (Table 3, model 2), respectively. However, even after controlling for relevant socio-demographic and work place characteristics as well as sleeping troubles, work-family conflict and supervisor support explained between 5 and 14% of additional variance in physical exertion (Table 4, model 3) and lack of motivation (Table 5, model 3).

## Discussion

This study aimed to test the association between work-family conflict and support from nearest superior and five different dimensions of work-related fatigue among Danish ferry crew members and terminal workers. We found that after controlling for potential confounders work-family conflict was consistently positively associated with fatigue, while higher supervisor support went along with lesser mental fatigue as well as less feelings of physical exertion. Further, our study also suggested that support from nearest superior can moderate a potential negative impact from work-family conflict on fatigue.

### The role of work-family conflict and support

Work-family conflict was significantly positively associated with four of the five sub-dimensions of fatigue, even after adjustment for general level of job demands. Thus, it is not the pure amount or tempo of work in the ferry shipping industry that counts, but also whether work obligations can be balanced with those from the family domain [1, 12, 57]. There can be different reasons for experiencing work-family conflict, such as long hours spent at work or non-flexible duty roosters [33, 35, 58, 59]. Whatever the individual reason, these employees may be particularly vulnerable to overextend their capacities when attempting to cope with conflicting challenges. And in doing so, they may be likely to experience frustration and feelings of guilt, anger or anxiety – all of which in the longer run may result in exhaustion and demotivation [60, 61].

To our knowledge, no other studies have investigated the role of work-family conflict for fatigue in ferry shipping or seafaring in general. However, the present findings are consistent with results from studies in other occupational fields. First, levels of work-family conflict and supervisor support are in line with those in other occupational groups [31]. Further, work-family conflict has been found to be a strong predictor for fatigue and for inter-shift recovery from fatigue among nurses’ aides and emergency and critical care nurses [7, 42]. Also, a meta-analysis showed that this factor was strongly associated with other work-related health indicators, such as burnout/exhaustion, or somatic/physical symptoms and psychological disorders (e.g. anxiety and depression) [61].

Notably, in our study, experience of work-family conflict alone seemed comparatively less relevant for the physical than the psychological sub-dimensions of fatigue, that is associations were higher and more consistent for the mental than the physical domain of fatigue. This may be explained by the fact that the physical fatigue scales of SOFI mainly tap into short-term symptoms, such as being out of breath or experiencing rapid heartbeat [22]. Such symptoms may mostly result from specific demands of manual labor [22], whereas work-family conflict may be more likely to manifest itself in terms of psychological symptoms [61], such as feeling drained, spent and/or worn out [22].

An independent protective effect was further identified for the resource factor ‘supervisor support’. Employees who perceived their supervisor as supportive were less likely to report lack of energy and lack of motivation. This is in line with evidence from research conducted in land-based occupational settings where support from supervisors has been consistently found to have a positive impact in cross-sectional [62] and longitudinal studies about fatigue [9, 62, 63]. Positive associations have further been reported from studies which have investigated the role of work-related support with respect to a range of other mental health-related outcomes than fatigue, such as anxiety and depression [28, 29, 47]. Such beneficial influences from supervisors may either result from feelings of being recognized and esteemed, or support is manifested in actual help with succesfully performing tasks [43].

However, it needs to be noted that effect sizes for supervisor support were mostly smaller than those for work-family conflict, suggesting that the stress factor in the present setting might have been stronger than the resource factor. Two further considerations, though, challenge this interpretation. First, it may be argued that an exclusive focus on the direct association between supervisor support and fatigue experience underestimates the potential contribution by supervisors. Arguably, supervisors might also help employees to reduce or even prevent the emergence of work-family conflicts in the first place by initiating practices to increase job control [64], especially control over work time [46] and work schedules [59, 65], for instance by considering individual needs and preferences in scheduling duty rota. To investigate such effects of supervisor support on work-family conflict, however, was not an aim of the present study, but would be an interesting focus for further research.

Second, the two interactions found between work-family conflict and supervisor support for the physical dimensions of fatigue indicate that support may alleviate negative consequences of work-family conflict, once present [34, 36, 40]. This is in accordance with the buffer hypothesis of the Demand-Control (−Support) model, which suggests that job-related resources, such as support from supervisors can mitigate adverse impact from high demanding or straining jobs [27–30, 32]. So even when conflicts between different domains of demands do occur – which to some extent may be unavoidable in work contexts such as ferry shipping - a resource like supervisor support can help to limit at least the physical manifestations of fatigue.

The fact that buffering occurred only for physical aspects of fatigue may be explained by two different mechanisms. Possibly, bodily manifestations of symptoms require a higher impact of stressful conditions, for instance simultaneous perception of work-family conflict and non-helpful supervisors, which might make those affected specifically vulnerable [34, 36]. Further, once work-family conflict is high supervisor support may be more effective in relieving physical work demands on board or at the terminals, for instance by allocating more resources for manual labor. It also needs to be noted that the interaction effects, while significant, were smaller than the main effects. Prior evidence from land-based occupational settings has suggested that the work stressor and the available coping resource should be matched for buffering to occur at all or to be substantial [28, 29]. Thus, the broader measures of work-family conflict and supervisor support used in the present study may have limited effect sizes or prevented detecting such associations for the mental aspects of fatigue.

It is important to note that subjective sleep quality was adjusted for in all analyses. In accordance with findings from a seafaring context [3, 4] as well as other occupational branches [13, 16, 55, 66, 67] sleeping troubles were indeed strongly associated with all aspects of fatigue. Thus, variance to be explained by the psychosocial factors under investigation was restricted. The present study only intended to investigate a direct pathway from perceived work environment to fatigue, which however does not exclude additional indirect or mediated effects of work-family conflict and supervisor support via sleep problems [13, 16, 55, 66, 67]. It could be argued that other sleep-related factors, such as sleep duration, and sleeping disorders, for instance sleep apnoea, might have been included as potential confounders. However, the COPSOQ-scale ‘sleeping troubles’ covers different aspects of sleep disturbance, such as ‘how often have you slept badly and restlessly?’ and ‘how often have you woken up too early and not been able to get back to sleep? [31]. Further, Danish seafarers must pass a medical examination each second year. It is therefore unlikely that crew members with medical conditions, such as severe sleeping disorders are represented in our sample. Terminal workers, however, are not required to engage in a similar medical screening program. Therefore, we cannot exclude that any of the participating terminal workers were suffering from a sleeping disorder.

### Limitations

As the cross-sectional design of the study did not allow for measuring change in fatigue over time, causal ordering could not be determined [68]. Therefore, we cannot differentiate to which extent the occurrence of more work-family conflict or lesser support from nearest superior preceded an increased experience of fatigue or if causation was reversed or reciprocal. The sole reliance on self-administered questionnaires for measuring fatigue and psychosocial variables exacerbates the problem of disentangling causation, since respondents with higher levels of perceived work-family conflict and less perceived support may have been more likely to report higher levels of fatigue or vice versa [68, 60]. Else, biases such as social desirability or a negativity bias as well as recall bias may have influenced ratings of both issues [68, 69].

All participants were assured confidentiality and could submit their responses without personal identification. Nevertheless, it cannot be excluded that some underreported fatigue or overreported supervisor support since high levels of fatigue or low levels of support from supervisors at work are considered problematic in general [70, 71]. Further, while it can be argued that the SOFI is psychometrically superior to the often used single-item measures of fatigue [21, 22], it was used only once and in retrospect, which might have led to over- or underestimation [20]. Also, respondents with more fatigue problems may have been more likely to report high levels of work-family conflict and/or lower levels of supervisor support due to recall bias.

A definite limitation is the modest response rate in Company 2 which, though common in a seafaring context [3, 4] and in some other branches of occupational research [5, 64], raises the question of sampling bias in terms of non-response/selective response [5, 70, 71]. In particular, the generalizability of the study’s findings is limited by the low participation rate in Company 2 and of those working in the terminals as these workers represent around one third of the employees within Danish ferry shipping companies [72]. Other comparisons with the overall population of employees in Danish ferry shipping, however, indicate no large deviations. Thus, most of the sample consisted of males of Danish origin in their late forties, half of who were officers, and for half of our sample typical worktime involved night shifts, all of which is in line with general characteristics of crew members working within Danish ferry shipping [72]. Other comparisons with the overall population of employees in Danish ferry shipping, however, indicate no large deviations. Further, an analysis for the employees of company 1, which contributed over 80% of the study participants, showed no significant differences between study participants and the total group of employees in terms of age, gender, occupational rank or workplace. This does not, however, exclude the possibility that those who felt more fatigued or those more critical towards their work environment may have been more likely to participate [70, 71]. Vice versa, since the study was endorsed by the shipping companies, it cannot be excluded that some employees – despite assurances to the contrary about anonymity or data protection - may have been reluctant to participate because they were hesitant to either share information about health complaints or negative ratings of their work environment [71]. Therefore, our sample may not be fully representative of the population, which raises questions about external validity. However, it should also be pointed out in this context that the evidence found for the work-family conflict and supervisor support interaction is in accordance with the body of research evidence from other areas of occupational research.

### Implications

For ferry companies our results indicate that it is not only work demands in general which should be considered [1, 4], but specifically demands with a high potential to conflict with employees’ family life [57, 61]. Therefore, ferry ship companies are encouraged to adopt organizational measures which reduce potential clashes, such as promotion of increased individual control over work time and -schedules [46, 59, 64, 65]. Further, to combat work-related fatigue among ferry ship employees, initiatives should be built around leader support, especially regarding work-family related issues. Such initiatives have been found to help counteract the negative impact of work-family conflict on health in onshore occupations [73]. It may, however, not be possible to completely avoid work-family conflict in a time-schedule regulated industry. Therefore, supervisor support appears to be a very important secondary strategy to buffer the potential consequences of work-family conflict as suggested by our finding that high level of support moderated the negative impact from conflict on certain aspects of fatigue (see also [27, 32, 74, 75].

However, to be able to make more specific recommendations about the design of fatigue intervention strategies more knowledge is needed. As a first step, studies designed to establish a clearer picture of the causal relationship between work-family and support from nearest supervisor and work-related fatigue in a ferry shipping (or seafaring) context are recommended. Such studies should be based on prospective longitudinal designs, including physiological measures of fatigue besides self-report assessments [68–71]. Furthermore, in such studies it would also be interesting to more closely investigate interactions and additive combinations between diverse types of fatigue-related risk factors in a ferry shipping (or seafaring) context [4]. Additionally, qualitative approaches may be used to identify the specific issues which characterize experience of work-family conflicts in ferry shipping as well as the specific actions by supervisors, which may provide effective buffering. Such qualitative approaches and/or quantitative once could also be applied to investigate potential advantages of working in the ferry shipping and/or seafaring industry. In a Danish context anecdotal evidence has pointed towards contractual tax benefits as an advantage together with work intensive tight scheduled work periods which are often compensated by longer continuous periods of off-work periods, but no systematic attempt has to our knowledge yet been taken to address this topic.

## Conclusion

The present study contributes to the scientific evidence on psychosocial work environment stressors and work-related fatigue. In particular, we have shown that it may be important to investigate subdimensions rather than a global or general sense of fatigue. In accordance with research findings from other occupational fields, we found that work-family conflict and support from nearest superior were potential influencing factors for work-related fatigue, particularly the mental aspects, in Danish ferry shipping. Our results further suggest that leadership support may offer a resource in moderating the negative impact from work-family conflict on the physical aspects of ferry ship employees’ fatigue. Based on our results ferry shipping companies are encouraged to center future fatigue preventive programs around work-family reducing initiatives, and that leader support may be a relevant component to include into such initiatives. However, to be able to make more specific recommendations about the design of such interventions, more studies are needed, investigating also other factors that potentially can buffer the negative effect from work-family conflict on fatigue.

## Data Availability

Data is available on request from the corresponding author.

## Abbreviations

COPSOQ: Copenhagen Psychosocial Questionnaire
LE: Lack of Energy
LM: Lack of Motivation
PD: Physical Discomfort
PE: Physical Exertion
S: Sleepiness
SOFI: Swedish Occupational Fatigue Inventory
